# Notes and News

**Published:** 1905-04-22

**Authors:** 


### Notes and News.

Friedenheim Hospital, Swiss Cottage, N.W., has received from the trustees of the Zunz Fund a donation of £3,000 for the purpose of maintaining a cancer ward of four beds to be named the "Annie Zunz" Ward.

We are asked to announce to our medical readers that medical men can be received at the Residence Universitaire, 95 Boulevard St. Michel, Paris, when visiting that city. The Université Hall has also a villa on the sea at Berck.

The late Mr. Luther Holden, who was a President of the Royal College of Surgeons, and Surgeon to St. Bartholomew's Hospital, has bequeathed £3,000 to that institution to found a "Luther Holden Scholarship in Surgery" and £500 to the Rebuilding Fund. He has also made the Benevolent Fund of the hospital residuary legatee for a larger sum.

At a meeting of the Royal Sanitary Institute at Liverpool Mr. F. Turton, Deputy-Surveyor to the Corporation of Liverpool, drew attention to the improvement in the sanitary condition of dwellings in Liverpool which had been effected during the past 40 years. In 1864 there were at least 22,000 houses structurally insanitary, whilst at the present time under 8,600 are defective in this way.

At the last meeting of St. George's Hospital, it was decided to proceed with the appeal for £350,000 to rebuild the hospital. At the same meeting the treasurer remarked that the grant made to the medical school was little more than must have been paid if the hospital had no medical school, but had to obtain house-physicians and house-surgeons and other officers who would be necessary.

The Lowestoft Hospital has had under consideration a change of name of the institution. "The North Suffolk Hospital" was suggested on the ground that patients are received from nearly the whole of the North Suffolk district, and that the alteration in the name might be the means of raising country subscriptions. The present subscribers' list is limited at only 500 out of a population of 50,000. The proposal was, however, abandoned, after much discussion, on the ground that the identity of the hospital would be lost. The advantages of the Hereford scheme of penny subscriptions was commented upon by the Chairman, who announced that it would probably be adopted in the near future in favour of the Lowestoft Hospital.

A correspondent suggests that the conditions of the "Speyer" competition apparently exclude stewards of hospitals and medical superintendents (whose appointment has been strongly advocated of late in these pages) unless they act as assistant secretaries.

The Jacksonian Prize for the year 1904 has been awarded by the Council of the Royal College of Surgeons of England to Mr. Herbert Paterson, F.R.C.S., assistant surgeon to the London Temperance Hospital, for his dissertation on "The Diagnosis and Treatment of such Affections of the Stomach as are Amenable to direct Surgical Interference."

A large deputation, organised by the Metropolitan Branch of the Incorporated Society of Medical Officers of Health, waited on the Managers of the Metropolitan Asylums Board to represent the desirability of the Board undertaking the provision of accommodation for metropolitan consumptives. The Chairman of the Board replied in sympathetic terms, and intimated that they were prepared to abide by the advice of the Local Government Board in the matter.

In the debate at the Hospital Officers' Club on Friday, the 14th inst., Professor MacHardy stated that he had enjoyed the unique privilege of building a hospital without incurring debt. In fact, as we pointed out at the time, the National Hospital for the Paralysed, Queen Square, a building at least four times as large as Mr. MacHardy's, was built and paid for before the opening, the money having been collected by labour wholly gratuitous. The same result was repeated in the case of the Finchley branch of the Queen Square Hospital, a building with the same number of beds—forty—as the South London Ophthalmic Hospital. Mr. Spurgeon's principle was to incur no liability for building until the money was in hand. That is a sound principle, and the instances quoted show, that it can be upheld in regard to voluntary hospitals when those responsible are wholeheartedly set on the attainment of the highest results.

The outbreak of small-pox originating on board the R.M.S. *Nile*, which arrived with one fatal case at Southampton on March 18th, has spread considerably since that date. There have been three deaths and about 30 cases under treatment. The infection is reported to have spread as far as Ireland, and one case is recorded at Hertford. The medical officer of the port maintains that all the precautions which were possible were taken. At present it appears that the authorities have no power to prevent persons leaving an infected ship if they are well at the time. This seems a mistake. The subject has been raised in the House of Commons, and it is to be hoped that the attention which has been drawn to the present defective regulations may effect a reform. In our opinion the medical officer of any port should have power to place a ship and all on board in immediate quarantine for a limited period, at any rate, until a report could reach the central authorities, who would confirm or rescind the order of the medical officer.

